# [Retracted] Hepatitis B core antigen regulates dendritic cell proliferation and apoptosis through regulation of PKC/NF-κB signaling pathway

**DOI:** 10.3892/mmr.2025.13651

**Published:** 2025-08-12

**Authors:** Lan Liu, Yanxin Huang, Kaili Zhang, Shupeng Song, Shuangxing Li, Yongguo Li, Yinghua Lan

Mol Med Rep 18: 5726–5732, 2018; DOI: 10.3892/mmr.2018.9604

Following the publication of this paper, it was drawn to the Editor's attention by a concerned reader that the four plots shown for the flow cytometric (FCM) data in Fig. 2 on p. 5728 contained overlapping areas, where differently performed experiments were intended to have been represented. Moreover, there were also indications that certain of the data were potentially overlapping with Figs. 4 and 5 (the other pair of figures containing FCM data in this paper).

Owing to the fact that the abovementioned data bore notable similarities comparing across different quadrants among these three figures, the Editor of *Molecular Medicine Reports* has decided that this paper should be retracted from the Journal on the grounds of an overall lack of confidence in the presented data. The authors were asked for an explanation to account for these concerns, but the Editorial Office did not receive a reply. The Editor apologizes to the readership for any inconvenience caused.

